# Baseline gut microbiome impacts probiotics *Bacillus licheniformis* CMCC63516 in modulating the gut microbiome and preventing antibiotic‐associated diarrhea: A double‐blind, randomized controlled trial

**DOI:** 10.1002/ctm2.1184

**Published:** 2023-04-05

**Authors:** Qian Zhou, Wenkui Dai, Yanmin Bao, Jing Chen, Xiaohua Han, Changshan Liu, Meijie Hou, Huisheng Yao, Changsuo Hao, Shuaicheng Li, Yuejie Zheng

**Affiliations:** ^1^ Department of Computer Science City University of Hong Kong Hong Kong China; ^2^ Department of Obstetrics and Gynecology Peking University Shenzhen Hospital Shenzhen China; ^3^ Department of Respiratory Diseases Shenzhen Children's Hospital Shenzhen China; ^4^ Department of Internal Medicine Shenyang Children's Hospital Shenyang China; ^5^ Department of Paediatrics The Second Hospital of Tianjin Medical University Tianjin China; ^6^ Department of Paediatrics Shenjing Hospital of China Medical University Shenyang China

**Keywords:** antibiotics‐associated diarrhea, children, double‐blind randomized controlled trial, gut microbiome, probiotics

Dear Editor,

Here, we report that baseline gut microbiome (GM) associate with the protection of probiotics against pediatric antibiotic‐associated diarrhea (AAD) via the double‐blind randomized control trial (RCT).

Antibiotics are widely prescribed drugs in infancy and childhood.^1^ Nevertheless, it may result in various adverse events such as AAD. Given the association of AAD with antibiotics‐induced GM changes,^2^, ^3^ recent studies demonstrated the protective role of probiotics in preventing pediatric AAD.^3^, ^4^, ^5^, ^6^ Nevertheless, there is no double‐blind RCT to assess the role of probiotics in AAD prevention for Chinese children. This study aimed to evaluate the protective effects of probiotics on pediatric AAD in China, based on multi‐center and double‐blind RCT (documented at www.chictr.org.cn under the accession number ChiCTR‐IPR‐16009033). In addition, we analyzed GM dynamics in response to antibiotics exposure and probiotics supplementation. The selected probiotics was Zhengchangsheng (*Bacillus Licheniformis* CMCC63516), which is a gram‐positive bacterium developed in China and has been proven effective in treatment of diarrhea.^6^

The inpatients with lower respiratory infections received antibiotic therapy for seven days, and *B. licheniformis* CMCC63516 (Group B) or placebo (Group P) was randomly administered (Supplementary File 1). Of 218 recruited patients, 173 children completed the trial (Group B, *n* = 87; Group P, *n* = 86) (Figure 1), and no adverse advents were reported. The AAD ratio was lower in Group B (6.90%) than that in Group P (11.63%) (Table S1). Metagenomic analyses were performed on 80 qualified feces samples for 40 children before and after the interventions (Group B, *n* = 11; Group P, *n* = 29) (Figure 1, Table S2). The raw data was submitted to CNGB Sequence Archive (CNSA) under project number: CNP0003013.

**FIGURE 1:**
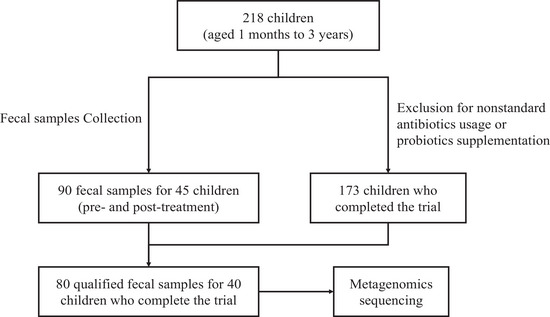
Sample inclusion in this study. A total of 218 children who fulfilled the requirements of the inclusion criteria were recruited from four hospitals. Subjects were excluded if (1) had diarrhea in recent weeks before enrollment; (2) were hospitalized in intensive care units; (3) had digestive tract malformation, digestive tract surgery, congenital heart disease, artificial heart membrane surgery, rheumatoid heart disease, or infective endocarditis history; (4) receiving immunosuppressant therapy, probiotic or Chinese medicine in recent weeks before enrollment. One hundred seventy‐three children received the one‐week intervention. Forty provided qualified fecal samples, which were collected before and after the intervention.

As measured by the Jensen‐Shannon distance,^7^ the baseline microbiota was clustered into three enterotypes, including cluster_B (dominated by *Bacteroides*), cluster_E (*Enterococcus*), and cluster_P_B (*Prevotella* and *Bacteroides*) (Figure 2A). The phylum Firmicutes was enriched in cluster_E, but Bacteroidetes decreased (Figure 2B). Additionally, the microbial samples in cluster_P_B had higher genus‐level bacterial diversity than the other two clusters (Figure 2C). Nevertheless, we found that microbial samples in the cluster_B had the highest inter‐individual dissimilarity at species level, followed by the cluster_E and cluster_P_B (Figure 2D). In consistence, the microbial samples in cluster_P_B had the most similar species‐level structures: predomination by *Prevotella copri*, *Bacteroides coprocola* and *Bacteroides plebeius*, followed by cluster_E (*Enterococcus faecium* and *Bacteroides vulgatus* represented the top two species) and cluster_B (dominated *Bacteroides* species varied dramatically) (Figure 2F, Table S2). Further analysis showed that the dissimilarity between the cluster_B and cluster_E was notably higher than that between the cluster_B and cluster_P_B, but the dissimilarity between cluster_E and cluster_P_B was the highest (Figure 2E).

**FIGURE 2:**
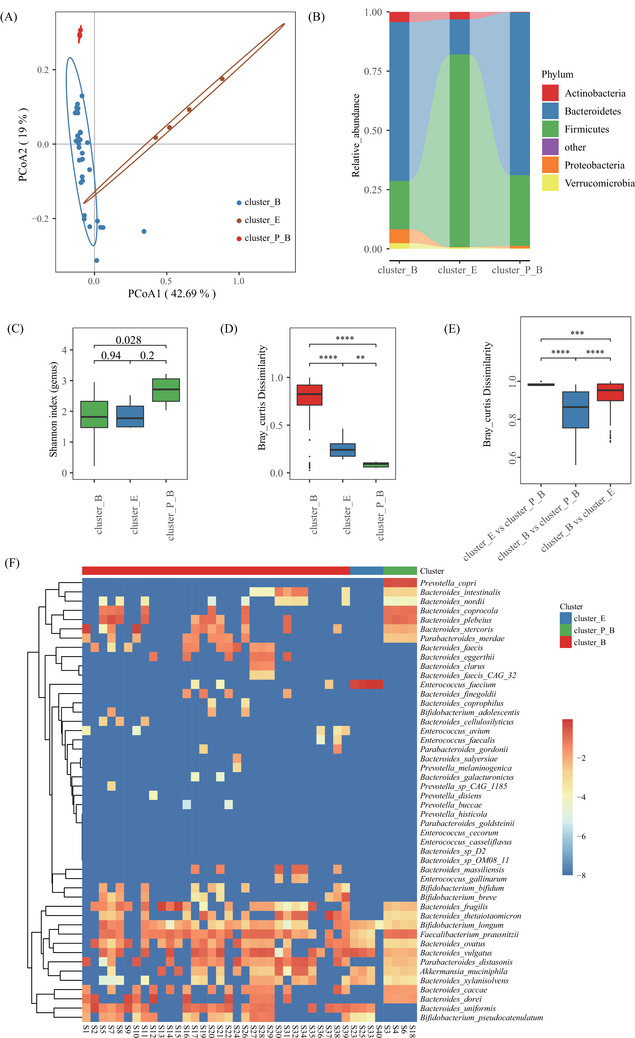
Inter‐individual structural variations for baseline gut microbiome (GM). (A) Principal coordinate analysis (PCoA) based on Jensen‐Shannon divergence as the distance measurement. (B) Distribution of phylum‐level GM compositions across three GM types. (C) Comparison of alpha‐diversity at genus level via the Shannon index. (D) Inter‐individual Bray‐Curtis distance for each type of GM. (E) Inter‐cluster Bray‐Curtis distance for three GM types. (F) Species‐level GM compositions for three GM types before clinical interventions and the colour in the heatmap represent log10 value of relative abundance. Wilcoxon rank‐sum test was applied to analyze the significance of inter‐cluster differences. **, ***, and **** represents adjusted *p*‐value <.01, <.001, and < .0001, respectively.

Permutational multivariate analysis of variance indicated that baseline GM structures and probiotic supplementation contributed to genus‐ and species‐level dissimilarity notably (at genus level, *p*‐value = .001 and .032 for baseline GM cluster and probiotics, respectively; at species level, *p*‐value = .001 and .053 respectively; Figure 3A,B). Further analysis found the lower GM variations of the cluster_B in response to clinical interventions as compared to the cluster_E and cluster_P_B (Figure 3C–E). In addition, the species‐level bacterial diversity decreased more sharply in cluster_E and cluster_P_B than in cluster_B (Figure 4A). We also found more significant changes of inter‐individual GM dissimilarity in the cluster_E and cluster_P_B (Figure 4B). And the GM dissimilarity between cluster_E and cluster_B/cluster_P_B notably reduced (Figure 4C). These findings suggested the higher GM stability of the cluster_B comparing to the cluster_E and cluster_P_B. This was partly consistent with the previous reports that *Bacteroides‐*dominated GM had high stability in the first three years of life,^8^, ^9^ and other enterotypes had a high probability of transition to the *Bacteroides*‐dominated enterotype.^9^

**FIGURE 3:**
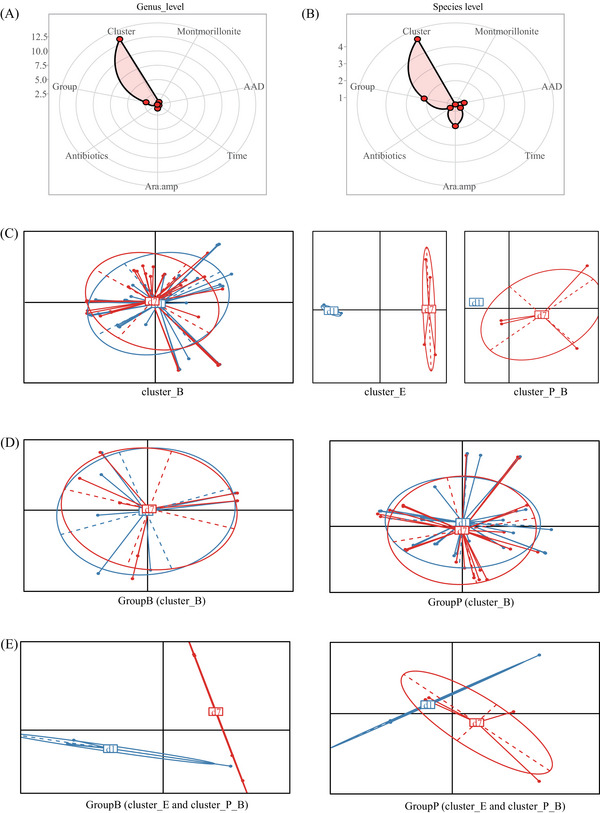
Factors notably impacting gut microbiome (GM) dynamics following clinical interventions. (A) Permutational multivariate analysis of variance (PERMANOVA) to assess the effects of several indices on genus‐level GM structures: the contribution is larger if the point is located near the outer circle. Montmorillonite: yes or no; AAD: yes or no; Time: d1 or d7; Ara.amp: yes or no; Group: Group B (probiotics) or Group P (placebo); Cluster: baseline GM types (cluster_B, cluster_E, or cluster_P_B). (B) PERMANOVA to assess the effects of several indices on species_level GM structures. (C) PCoA based on the Bray‐Curtis distance between d1 and d7 microbial samples of each patient. GM changes for cluster_E and cluster_P_B are larger than those for cluster_B. (D) For patients with the baseline cluster_B GM type, PCoA based on the Bray‐Curtis distance between d1 and d7 microbial samples of each individual. The dissimilarity in Group P is slightly higher than that in Group B. (E) For patients with the baseline non‐Bacteroides‐dominated GM type, PCoA based on the Bray‐Curtis distance between d1 and d7 microbial samples of each individual. The dissimilarity in Group B is slightly higher than that in Group P.

**FIGURE 4:**
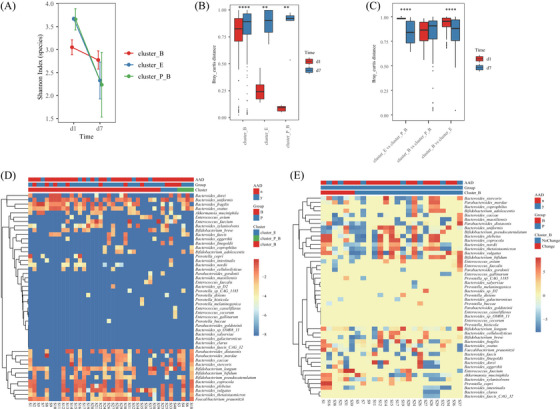
Changes in species‐level GM for individuals with different baseline gut microbiome (GM) structures. (A) Decreased alpha diversity for each GM type after clinical interventions. (B) Differences of inter‐individual Bray‐Curtis distance between d1 and d7 timepoints in each GM type. (C) Differences of inter‐group Bray‐Curtis distance between d1 and d7 timepoints. (D) Species‐level GM compositions for three GM types after clinical interventions and the colour in the heatmap represent the log10 value of relative abundance. (E) Species‐level GM structural changes for microbial samples with baseline Bacteroides‐dominated GM type and with placebo. The colour in the heatmap represents the log10 value of (d7 abundance/ d1 abundance) for each species. Wilcoxon rank‐sum test was applied to analyze the significance of inter‐group differences. ** and **** represent adjusted *p*‐value < .01 and < .0001, respectively.

To better understand how GM changes differed with baseline GM structures and probiotic interventions, we applied penalized generalized estimating equations^10^ to analyze the main genus‐level contributors to GM dynamics. We observed that GM changes were mainly associated with the level of *Akkermansia*, *Bacteroides*, *Bifidobacterium*, *Enterococcus* and *Faecalibacterium*. Given the high levels of *Parabacteroides* and *Prevotella* in several samples, we analyzed time‐serial bacterial changes in the seven genera mentioned above. For both probiotics and placebo, GM varied in cluster_E and cluster_P_B, that is, dramatically reduced levels of *E. faecium* and *P. copri* that dominated at baseline, as well as elevated levels of distinct *Bacteroides*, *Bifidobacterium*, and *Faecalibacterium* species (Figure 4D). The change of Kyoto Encyclopedia of Genes and Genomes database (KEGG) pathway abundance also showed that GM functional variations differed with baseline GM clusters (Figure S1, Supplementary File 1). This further suggested the robust impact of interventions on the cluster_E and cluster_P_B GM structures.

Nonetheless, notable structural GM variations also existed for subjects in cluster_B, including S1, S16, S21, S31, S38, and S39, who all received placebo supplementation (Figure 4D,E). The predominance of the GM components for subject S1 and S16 dramatically changed from *Bacteroides* species to *P. copri* (from 10e‐8 to 36.92% and 36.41% respectively), *B. coprocola* (from 10e‐08 to 10.16% and 9.25%) and *B. plebeius* (from 10e‐8 to 9.26% and 10.49%) (Figure 4D,E, Table S2). Consistently, we observed a positive correlation between *P. copri* and *B. coprocola*, as well as *B. plebeius* (*r* > .3, adjusted *p*‐value < .05, Figure S2). For subject S38, *Bifidobacterium longum* represented the highest abundance after clinical treatment (from 5.62% to 81.52%) (Figure 4D,E, Table S2). And *E. faecium* increased to be the predominant species for subject S21, S31 and S39 (from .01%, 10e‐08, 10e‐08 to 56.31%, 70.18% and 99.79% respectively) (Figure 4D,E, Table S2). Further analysis did not observe significant differences in age, baseline GM diversity, and AAD incidence among six samples (Table S2).

The limitations of our study are the small sample size and non‐uniform antibiotics. It may bias statistical significance to the protective role of probiotics. Nonetheless, this study is the first double‐blind RCT to assess the role of probiotics in AAD prevention and observes the impact of pre‐existing GM structures on clinical efficacy of probiotics for Chinese children.

In conclusion, this study suggests the impact of preexisting GM on the protection of probiotics against AAD and implies the necessity of considering preexisting GM structures when assessing clinical efficacy of probiotics in AAD prevention.

## CONFLICT OF INTEREST

The authors declare that there is no conflict of interest that could be perceived as prejudicing the impartiality of the research reported.

## FUNDING INFORMATION

Shenzhen Key Medical Discipline construction Fund, Grant Number: SZXK032; Scientific Research Foundation of Peking University Shenzhen Hospital, Grant Number: KYQD2021075

## Supporting information

Supporting Information

Supporting Information

Supporting Information

Supporting Information

Supporting Information
