# Supplementary material for: Baseline gut microbiome impacts probiotics Bacillus licheniformis CMCC63516 in modulating the gut microbiome and preventing antibiotic‐associated diarrhea: A double‐blind, randomized controlled trial
Source: Clin Transl Med. 2023 Apr 5;13(4):e1184. doi: 10.1002/ctm2.1184 (PMC10076687; doi:10.1002/ctm2.1184)
Supplement: Supplementary file 1 — Supporting Information [file CTM2-13-e1184-s004.docx]

**Supplementary information**

**Ethical statement.** The double-blind RCT was registered at [www.chictr.org.cn](http://www.chictr.org.cn) under the number ChiCTR-IPR-16009033. This study was approved by the Ethics Committee of Shenzhen Children’s Hospital, The Second Hospital of Tianjin Medical University, Shenjing Hospital of China Medical University and Shenyang Children’s Hospital.

**Study design, sample collection, and DNA extraction.** The inclusion criteria for the enrollment of the patient were as follows: aged one month to three years; no diarrhea; inpatients diagnosed with lower respiratory infection; receiving antibiotic therapy 7-14 days after hospitalization. Subjects were excluded according to the following criteria: having diarrhea in recent weeks before enrollment; hospitalized in intensive care units; having digestive tract malformation, digestive tract surgery, congenital heart disease, artificial heart membrane surgery, rheumatoid heart disease or infective endocarditis history; receiving immunesuppressant therapy, probiotic supplementation or Chinese medicine in recent weeks before enrollment.

The probiotics *B. licheniformis* CMCC63516 (*Bacillus licheniformis* Zhengchangsheng®, China) or placebo, which was randomly assigned by a thirty-party scientist, were administered to the included patients. Both doctors and patients did not know if the product was probiotics or placebo. Patients had probiotics or placebo supplementation as the followed protocol: once for 0.25 g powder and three times a day for ≥6 days. AAD was diagnosed according to the followed criteria: increased defecation frequency (at least twice/days; for example, 3 times/day at enrollment and increased to 3-5 times/day after clinical interventions) and changed stool shape (from Bristol stool type 1-6 at enrollment to type 7 after treatment).

Feces were collected at enrollment and after seven days of treatment, preserved in a 2 ml sterile tube at 4 centigrade and then stored at -80 centigrade 30 minutes after collection. Microbial DNA was extracted by the cetyltrimethylammonium bromide method. The quality of the PCR products was verified by Qubit4.0 (Thermo Fisher Scientific, Singapore). Sequencing libraries were generated using the NEBNext® UltraTM DNA Library Prep Kit (NEB, USA). The qualified DNA libraries were then performed with 2x150bp paired end sequencing via the Illumina NovaSeq 6000 platform (Illumina, San Dieago, CA, United States).

**Metagenomic sequencing and data processing.** The raw sequencing reads were filtered to remove low-quality sequences, library primers, and adapters using trimmomatic (v0.39.2) with default parameters.^1^ The host DNA sequences were then removed by mapping filtered reads to the human genome database (version hg38) by bowtie2 (v2.3.5.1). The high-quality unmapped reads were annotated via MetaPhlAn3 (v3.0.13).^2^ Profiling tables of the read counts for each genus and species were applied to calculated microbial diversity at the genus and species level, respectively, based on the Qiime2 package (2021.11).^3^ The taxon, which presented in at least ten samples and exceeded 0.5% average relative abundance, was selected for further analysis.

**Statistic analysis.** We selected genus and species that represented 0.01% abundance in at least one subject for further analysis. Pretreatment microbial samples were clustered based on the Jensen-Shannon distance, as previously reported.^4,5^ The impact of different indices on GM distributions was assessed by Permutational Multivariate Analysis of Variance (PERMANOVA) with 9,999 permutations (package *vegan* in R). The Bray-curtis distance, which was applied to assess the dynamics of GM after clinical interventions for each patient, was calculated using the *adonis* function in the vegan R package. The R package *nlme* and *lmerTest* was utilized to evaluate xwhether baseline GM affected the Bray-Curtis distance between pre- and post-treatment for each subject. Independent fixed effect variables included the top three principal components for baseline genus-level GM, baseline Shannon index, antibiotics types, use of Ara.amp and Montmorillonite, AAD incidence. The enrollment day and the hospital site were selected as random effects. Penalized Generalized Estimating Equations (package *PGEE* in R) was used to analyze the contribution of baseline GM components at the genus level to GM changes (bray-curtis distance for each patient).^6^ Analysis results were visualized via R software (version 4.0.3). Wilcoxon rank-sum test was applied to analyze the significance of inter-group differences and the p value was adjusted by the Benjamini & Hochberg method.

**Author’s contributions.** Y.Z. and S.L. managed the project and guided data interpretation. Q.Z. and W.D. performed the bioinformatics analysis, mined the data and wrote the manuscript. Y.B., J.C. and X.H. collected samples and clinical information. C.L., M.H. and C.H. conducted the sequencing experiment, optimized the graphs and data curation. S.L. and Y.Z. polished the manuscript.

**Reference**

1 Bolger, A. M., Lohse, M. & Usadel, B. Trimmomatic: a flexible trimmer for Illumina sequence data. *Bioinformatics* 2014; **30**, 2114-2120

2 Beghini, F., McIver, L. J., Blanco-Míguez, A. *et al.* Integrating taxonomic, functional, and strain-level profiling of diverse microbial communities with bioBakery 3. *Elife* 2021; **10**

3 Bolyen, E. A.-O., Rideout, J. A.-O., Dillon, M. A.-O. *et al.* Reproducible, interactive, scalable and extensible microbiome data science using QIIME 2. *Nat Biotechnol* 2019; **37**, 852-857

4 Arumugam, M., Raes, J., Pelletier, E. *et al.* Enterotypes of the human gut microbiome. *Nature* 2011; **473**, 174-180

5 Koren, O., Knights D Fau - Gonzalez, A., Gonzalez A Fau - Waldron, L. *et al.* A guide to enterotypes across the human body: meta-analysis of microbial community structures in human microbiome datasets. *PLoS Comput Biol* 2013; **9**, e1002863

6 Wang, L., Zhou, J. & Qu, A. Penalized generalized estimating equations for high-dimensional longitudinal data analysis. *Biometrics* 2012; **68**, 353-360

**Supplementary Figure Legends**

Fig S1. The alteration of gene functional profile at KEGG Pathway level. The color ranged from blue to red represents the log2 value of ratio (d7 abundance to d1 abundance) for each pathway.

Fig S2. Correlation network for bacterial species. The size of circle represents the number of connection. The blue line represents negative correlation (r < -0.3, p value > 0.05) and the red line represents positive correlation (r > 0.3, p value < 0.05).
