# Supplementary material for: Baseline gut microbiome impacts probiotics Bacillus licheniformis CMCC63516 in modulating the gut microbiome and preventing antibiotic‐associated diarrhea: A double‐blind, randomized controlled trial
Source: Clin Transl Med. 2023 Apr 5;13(4):e1184. doi: 10.1002/ctm2.1184 (PMC10076687; doi:10.1002/ctm2.1184)
Supplement: Supplementary file 4 — Supporting Information [file CTM2-13-e1184-s001.docx]

Table S1. Sample information for enrolled subjects

|  | ***Bacillus licheniformis* CMCC63516 (Group B, n=87)** | **Placebo**  **(GroupP, n=86)** |
| --- | --- | --- |
| **Age (d)** | 408(60-1080) | 408(30-1080) |
| **Gender** |  |  |
| Female | 35(40.23%) | 28(32.56%) |
| Male | 52(59.77%) | 58(67.44%) |
| **Delivery** | | |
| VD  CS  Unknown | 45(51.72%) | 33(38.37%) |
|  | 42(48.28%) | 46(53.49%) |
|  | 0(0%) | 7(8.14%) |
| Full term | 81(93.10%) | 79(91.86%) |
| **Feeding pattern** | | |
| Human milk | 43(49.43%) | 42(48.84%) |
| Mixed | 33(37.93%) | 34(39.53%) |
| Formula | 9(10.34%) | 9(10.47%) |
| Unknown | 2(2.30%) | 1(1.16%) |
| **Antibiotics** | | |
| Penicillin | 17(19.54%) | 22(25.58%) |
| Cephalosporin | 55(63.22%) | 54(62.79%) |
| Macrolide | 6(6.90%) | 3(3.49%) |
| Penicillin + Macrolide | 1(1.14%) | 1(1.16%) |
| Cephalosporin + Macrolide | 8(9.20%) | 6(6.98%) |
| **Other drugs** | 77(88.51%) | 73(84.88%) |
| **AAD** | 6(6.90%) | 10(11.63%) |
